# The Glymphatic System in Diabetes-Induced Dementia

**DOI:** 10.3389/fneur.2018.00867

**Published:** 2018-10-31

**Authors:** Young-Kook Kim, Kwang Il Nam, Juhyun Song

**Affiliations:** ^1^Department of Biochemistry, Chonnam National University Medical School, Gwangju, South Korea; ^2^Department of Biomedical Sciences, Center for Creative Biomedical Scientists, Chonnam National University, Gwangju, South Korea; ^3^Department of Anatomy, Chonnam National University Medical School, Gwangju, South Korea

**Keywords:** glymphatic system, diabetes-induced dementia, sleep, cognitive decline, norepinephrine

## Abstract

The glymphatic system has emerged as an important player in central nervous system (CNS) diseases, by regulating the vasculature impairment, effectively controlling the clearance of toxic peptides, modulating activity of astrocytes, and being involved in the circulation of neurotransmitters in the brain. Recently, several studies have indicated decreased activity of the glymphatic pathway under diabetes conditions such as in insulin resistance and hyperglycemia. Furthermore, diabetes leads to the disruption of the blood-brain barrier and decrease of apolipoprotein E (APOE) expression and the secretion of norepinephrine in the brain, involving the impairment of the glymphatic pathway and ultimately resulting in cognitive decline. Considering the increased prevalence of diabetes-induced dementia worldwide, the relationship between the glymphatic pathway and diabetes-induced dementia should be investigated and the mechanisms underlying their relationship should be discussed to promote the development of an effective therapeutic approach in the near future. Here, we have reviewed recent evidence for the relationship between glymphatic pathway dysfunction and diabetes. We highlight that the enhancement of the glymphatic system function during sleep may be beneficial to the attenuation of neuropathology in diabetes-induced dementia. Moreover, we suggest that improving glymphatic system activity may be a potential therapeutic strategy for the prevention of diabetes-induced dementia.

## Introduction

The relationship between type 2 diabetes mellitus (T2DM) and dementia, also called “type 3 diabetes”, has emerged as a critical health issue across the world as it is fast increasing in incidence (1). There are several studies supporting the relationship between diabetes and dementia. A recent meta-analysis indicated that individuals with T2DM have a 65% increased risk for Alzheimer's disease (AD) (2). A population-based longitudinal study has reported a 16% increased risk for dementia in T2DM patients compared with nondiabetic patients (3). Furthermore, many T2DM patients have cognitive impairments (4) and even patients in the prediabetic stage of insulin resistance exhibit a decrease in memory function and dysfunction of cognitive flexibility and cognitive control (5). Moreover, patients with chronic hyperinsulinemia exhibit insulin resistance and cognitive dysfunction (6). Taking into consideration the previous evidence for the relationship between diabetes conditions, including insulin resistance and hyperglycemia, and cognitive decline (5), the neurological changes in diabetes-induced dementia should be investigated to elucidate the underlying mechanisms and thus support the design of appropriate therapy and prevention in clinical practice.

Current studies highlight the role of the glymphatic system in neurodegenerative diseases such as AD, suggesting that it influences the clearance of amyloid-beta (Aβ) peptide (7). In addition, the roles of the glymphatic system in the central nervous system (CNS) include the regulation of astrocyte activity (8), neurohormones (9), and glucose metabolism (10); modulation of apolipoprotein E (APOE) circulation (11); and regulation of insulin resistance in the brain (12, 13). Diabetes has been shown to contribute to the impairment of glymphatic activity, leading to declined cognitive function (14). Sleep has been known to promote the activity of the glymphatic pathway, subsequently enhancing the efficiency of the brain-clearance system (15), and ultimately improving memory function and synaptic plasticity (16). Here, we have reviewed recent evidence for the relationship between glymphatic pathway dysfunction and diabetes-induced dementia. Furthermore, we have suggested a therapeutic approach for alleviating the neuropathological symptoms of diabetes-induced dementia through the improvement of glymphatic system function.

## What is the glymphatic system?

In the CNS, approximately 68% of the total water volume is within the intracellular compartment, whereas the remaining 32% of the water exists in the extracellular compartment (8). The extracellular fluid is distributed into the interstitial fluid (ISF), cerebrospinal fluid (CSF), and blood compartments (8). In the CNS, a variety of nutrients circulate through the brain, but it is also essential to remove efficiently the metabolites (17). The CSF produced by the choroid plexus contributes to the delivery of nutrients to the brain parenchyma and the clearance of interstitial toxic waste (18, 19). The CSF is absorbed inside the CNS via filtration and reabsorption of water through the capillaries into the ISF of the surrounding brain regions (20, 21). The CSF enters the para-arterial spaces, mixes with the ISF, and is finally removed from the brain through the paravenous spaces (18, 22). The ISF with the toxic peptide wastes enters the lymphatic circulation through the paravenous space (7, 23). The ISF drained into the paravenous space can eliminate the solutes from the interstitial space similar to the function of the lymphatic system outside the brain (18).

The perivascular space of cerebral blood vessels is the place where the CSF/ISF exchange occurs (18, 24). In addition, the glial cells that provide the outer boundary of the perivascular space have an important function in the clearance and waste turnover (25, 26). Iliff et al. demonstrated that fluorescent dextran injected into the cisterna magna in mice was found in the basement membrane of parenchymal capillaries and in the perivascular spaces of caliber draining veins. This paravascular clearance system was named as the glymphatic system by merging the words “glial” and “lymphatic”, because of its dependence on glial cells and also due to its functional resemblance to the peripheral lymphatic system (18). In other words, the glymphatic system is a paravascular pathway that lies in the space between the vascular adventitia and the vascular end-feet of astrocytes (27). The glymphatic system has greater vascular permeability and contributes to the clearance of macromolecules from the brain parenchyma (27). Moreover, the glymphatic pathway contributes to CSF influx into the brain parenchyma through para-arterial spaces to exchange solutes with the ISF (28). Previous studies about the glymphatic pathway have fundamentally altered the traditional model of CSF hydrodynamics (18, 29) and shown that CSF can be recycled back into the brain and exchanged with ISF (29–31). Current studies have reported that the glymphatic pathway involves the dural lymphatic vessels that finally drain toward the cervical lymph nodes (23, 32, 33).

The glymphatic pathway has important diverse roles. Some studies have highlighted the role of the glymphatic pathway in waste drainage. Indeed, it was demonstrated that the glymphatic pathway could be the first step of the brain drainage system (34). Another study reported that the CSF containing toxic waste circulates in the arachnoid space and flows to the dural venous sinuses (7, 35). Moreover, the role of the glymphatic system in nutrient delivery was reported by demonstrating that lipoproteins and small molecules could be delivered from the CSF to the brain parenchyma via the glymphatic pathway (36). Other researchers have shown that the glymphatic system is crucial for the distribution of nutrients throughout the whole brain (37, 38). Additionally, several studies identified the role played by the glymphatic pathway in hormone circulation and signal transduction. It was reported that the glymphatic pathway is not only involved in the volume transmission and the paracrine system, but also in the activation of astrocytic Ca^2+^ signaling within the cortex (30) and in the opening of *N*-methyl-d-aspartate (NMDA) receptors in cultured astrocytes (39). Furthermore, the glymphatic pathway could be involved in regulating the circulation of norepinephrine, the major neuromodulator of arousal (40) that is related with cognitive decline (9), and which plays a role in AD neuropathology (41). Moreover, the glymphatic pathway influences not only the CNS, but also other organs via the circulatory system (42). In this view, current anatomical studies support the fact that the glymphatic pathway is connected with the peripheral system through glymphatic efflux sites, including arachnoid granulations, perineural spaces of cranial and spinal nerves (43), and meningeal lymphatics (23). Based on these observations, the function of the glymphatic pathway is deemed indispensable, and further studies are necessary for the identification of its relationship with various neurological changes.

## Glymphatic system dysfunction and alzheimer's disease

As mentioned earlier, the glymphatic system acts as an effective waste-clearance pathway for the brain (28). Previous studies demonstrated that dysfunctions of the glymphatic system aggravate neuropathological symptoms of various neurological diseases such as stroke and AD (8, 44). One magnetic resonance imaging (MRI) study indicated that alterations of the glymphatic system could be used as disease risk indicators for neurodegenerative disorders, including AD (45). Impairment of the glymphatic pathway can be the result of abnormal changes in CSF influx dependent on arterial pulsatility (8, 29). The AD is characterized by several neuropathologies, including Aβ accumulation and the tau tangle formation in various brain regions (46). Blood-brain barrier (BBB) breakdown increases the accumulation of Aβ in the blood plasma, ISF, and CSF (47), causing synaptic dysfunction in the brain. Furthermore, BBB disruption causes inflammation that also contributes to glymphatic dysfunction, suppresses CSF-to-ISF turnover, and impairs glymphatic clearance (48, 49). Amyloid-beta peptide exists in the normal brain, circulating blood, and CSF (50). While the normal brain is able to control Aβ influx and efflux through glymphatic drainage, the AD brain cannot control this process. Therefore, toxic Aβ accumulates in the brain parenchyma and vascular structures (51), and ultimately triggers BBB disruption and vasculature impairment (16).

Amyloid-beta clearance via BBB transport depends on the glymphatic pathway (52), as toxic Aβ can be transported across the BBB through specific transporters such as the low-density lipoprotein receptor-related protein-1 (53). However, when the amount of Aβ exceeds the capacity of the efflux transporter, Aβ is cleared via ISF flow in the glymphatic system (54, 55). The glymphatic system drains over 60% of the brain Aβ to the lymph nodes using the convective flow caused by arterial pulsations (56). The increased permeability of the BBB triggers glymphatic pathway impairment and ultimately leads to the defective clearance of Aβ by BBB transport in dementia (42, 57). Thus, the glymphatic system is considerably relevant to AD progression by transporting Aβ and other metabolites out of the brain (15).

The dysfunction of the glymphatic pathway increases the accumulation of toxic waste products in the brain (58), and is associated with impaired cognitive function recorded in behavioral tests (59, 60). Moreover, it is associated with the dysregulation of water transport into astrocytes (8). Aquaporin-4 (AQP4), a water transport channel expressed in the astrocytic end-feet near the capillaries, is considered to be critical for water movement between the cellular and ventricular compartments (61). Loss of AQP4 results in impairment of CSF influx and CSF-to-ISF turnover (62), aggravating glymphatic pathway dysfunction (28). The loss of AQP4 polarization has been related to glymphatic dysfunction in the brains of mice and considered to be a predictor of AD in humans (63). Additionally, the decrease in AQP4 expression contributes to reduced Aβ clearance (63–65) and tau clearance (66) through the glymphatic system (67), and has been shown to impair water permeability *in vitro* (68). Moreover, AQP4 is associated with the modulation of neurotrophic factor-dependent synaptic plasticity (69), and its absence results in defects in memory consolidation (70, 71). Collectively, the glymphatic system is affected by AQP4 expression in astrocytes and associated with AD progression.

Furthermore, a recent study suggested that an increase in CNS norepinephrine levels and ISF secretion are the results of reduced glymphatic influx in AD mouse models (9). Noradrenergic neurons located in the locus coeruleus supply norepinephrine to various brain regions (72). Elevated norepinephrine levels result in the contraction of the extracellular volume fraction, reduction of CSF influx, and brain ISF (15). Locus coeruleus-derived norepinephrine increases BBB permeability by elevating Na^+^/K^+^-ATPase activity, leading to augmentation of ISF secretion, and subsequently contributing to the glymphatic function (73). The noradrenergic system in the brain has critical roles in cognitive activities, including attention, perception, and memory function (41, 74). Loss of locus coeruleus neurons and abnormal levels of CSF norepinephrine were observed in the AD brain (75, 76). In addition, several subtypes of adrenergic receptors have been shown to control the production of Aβ (77) or mediate Aβ toxicity (78), involved in AD pathogenesis. Altogether, norepinephrine contributes to the function of the glymphatic system and is implicated in the neuropathology of AD including memory loss.

The *APOE* gene, the only strongly confirmed genetic risk factor for AD, has been associated with cognitive impairment (79), lipid metabolism, and various brain pathologies (80). The CSF is a major source of *APOE* for ISF because it circulates through the brain parenchyma via the glymphatic pathway (15, 81). The CSF contributes to the delivery of APOE to the brain via the glymphatic system, for molecules including Aβ (9), lipophilic molecules (30), and tau (82). Apolipoprotein E has been known to regulate transport and metabolism of cholesterol in the periphery and CNS (83), and is further associated with neurite growth, synaptic plasticity, and cognitive function (84). The *APOE* polymorphisms influence the structure and function of the glymphatic pathway (85) and there is a strong correlation between the ε*4* allele and neurodegeneration (86).

In conclusion, dysfunctions of the glymphatic pathway and subsequent impairment of metabolite circulation aggravate the onset and development of AD. Further studies on the implication of the glymphatic system in AD are necessary for the development of effective therapeutic strategies for AD.

## The glymphatic system and diabetes-induced dementia

Diabetes has been known to be a risk factor for various complications including hypertension, cardiovascular diseases, and neurological diseases, such as stroke and AD (87, 88). Recently, diabetes-induced dementia has been highlighted in CNS studies, showing that diabetes features, including insulin resistance and hyperglycemia, can trigger impairment of memory function, neuronal cell damage, and neuroinflammation (18, 89–91). A recent study demonstrated synaptic dysfunction through the loss of synaptic proteins in hyperglycemia-induced dementia (92). Moreover, in line with the diagnosis of diabetes in patients with cortical embolism due to atherothrombosis and stroke, diabetes conditions may alter the arteriolar structure and influence the perivascular space in the brain (93). A previous study demonstrated that Aβ plaques were accumulated in the brains of diabetes patients affecting cognitive function (94). Other experimental studies showed that diabetes induced by high-fat and/or high-sugar diets triggered Aβ accumulation in the brain (95–98). Therefore, more study on the mechanisms linking diabetes and dementia is necessary for understanding of the onset and progression of diabetes-induced dementia.

Type 2 diabetes mellitus is characterized by enhanced glymphatic CSF influx and a slowing of the interstitial solute clearance, leading to cognitive decline (13). Several studies have reported diabetes-associated cerebrovascular dysfunctions, neurodegenerative processes, and cognitive impairments following abnormal glycemia and insulinemia (5, 10, 12, 99). Another study has reported that hyperglycemia could result in cerebral neurovascular dysfunction, neurotoxicity, and impairment of neural insulin metabolism, leading to cognitive impairment (100). Chronic microvascular dysfunction caused by hyperglycemia can also cause severe cognitive dysfunction in diabetes patients (5, 10, 99).

Moreover, diabetes is associated with vascular pathology. It contributes to the development of small blood vessel disease and triggers the impairment of glymphatic activity, leading to cognitive dysfunction (14, 101, 102). Several studies have shown that BBB integrity was compromised and permeability was dramatically increased in the brain of diabetes patients (103). The BBB protects the brain against toxic components that may cause synaptic dysfunction or generate neurotoxins and maintains homeostasis (104, 105).

Hyperglycemia caused by diabetes is also related to neuronal pathogenesis by inducing the generation of excessive reactive oxygen species (ROS) and microvascular complications (106) and by suppressing the supply of vitamin C as an antioxidant and scavenger of free radicals into the brain, subsequently promoting oxidative stress in the brains of diabetes patients (107, 108). In addition, diabetes results in abnormal cerebral neovascularization and neurovascular remodeling (109). Brain endothelial cells are vulnerable to hyperglycemic stress in diabetes (110) and diabetes-induced hyperglycemia is associated with neurodegenerative diseases such as AD (111). Hyperglycemia promotes the generation of excessive superoxide species and boosts the activation of the protein kinase C (PKC) and advanced glycation end products (AGE) pathway, leading to increased BBB permeability mediated by the disruption of tight junction proteins and increased vascular endothelial growth factor (VEGF) expression (112, 113).

Moreover, one study demonstrated that the correlation between diabetes and AD depends on the *APOE*ε*4* allele, which was involved in lipid homeostasis in diabetes (114). In diabetes patients, the increase of *APOE*ε*4* increases the risk for AD compared with nondiabetic patients (115).

In conclusion, diabetes triggers the disruption of the BBB and increase of APOE and ultimately aggravates cognitive decline through metabolite imbalance due to glymphatic pathway dysfunction. Thus, the investigation and the understanding of the role of the glymphatic system in diabetes-induced dementia are necessary for the development of an efficient treatment for diabetes-induced dementia.

## The importance of sleep in diabetes-induced dementia

Sleep is necessary for the bulk flow of brain ISF and clearance of solutes, and is also involved in memory function and synaptic plasticity through several mechanisms, including Ca^2+^/calmodulin-dependent protein kinase II (CaMKII) signaling (16, 116–118). It was reported that astrocytes undergo contraction during sleep, and subsequently, the extracellular space is enlarged and the flow of ISF is enhanced. These processes promoted the clearance of macromolecular metabolites from the brain, during sleep (15). The disturbance of glymphatic transport due to inadequate sleep may mediate neuropathologies in AD, given that sleep disruption aggravates the assembly of Aβ plaques and tangles (119, 120). Recent studies have focused on the effect of sleep deprivation on synaptic plasticity and on structural changes of brain regions related to learning and memory (121, 122), and the progression of AD (123–125). Moreover, impaired glymphatic transport results in a 40% decrease in the clearance of Aβ in the brain of mice (54). Sleep disturbance is also associated with the deterioration of diabetes conditions such as insulin resistance (126), the dysregulation of energy and glucose homeostasis in healthy adults (127, 128), and the dysregulation of body weight (129). Thus, the influence of sleep on glymphatic transport is an important aspect of manipulation to control glymphatic system dysfunction in diabetes-induced dementia.

During sleep deprivation, norepinephrine secretion is increased (130), while glymphatic fluid transport is reduced (131). Diabetes conditions, such as hyperglycemia, also cause changes in the CSF concentrations of norepinephrine (132–134). Norepinephrine results in vasoconstriction of the pial arteries (135) and leads to the reduction of CSF inflow during sleep deprivation (9). In addition, several studies have demonstrated that the impairment of glymphatic pathway activity caused by sleep deprivation triggered APOE-related neuronal dysfunction in AD, leading to cognitive decline (11, 16). Hence, the modulation of norepinephrine secretion and other related pathways may enhance the function of the glymphatic system and ameliorate memory in diabetes-induced dementia.

Sleep is influenced by the hormone melatonin, which is mainly produced in the pineal gland, which receives input from the suprachiasmatic nucleus in the hypothalamus (136, 137). Melatonin is the major hormone regulating the circadian rhythm (138) and is also known to regulate memory function by acting on hippocampal neurons involved in memory formation (139, 140). Several studies have reported that melatonin could control hippocampal synaptic plasticity by binding to the melatonin specific receptor (141) and alter synaptic transmission and long-term potentiation in the hippocampus (142). In addition, melatonin could regulate calcium influx by controlling the conductance of voltage-gated Ca^2+^ ion channels and NMDA receptors (143, 144) in gamma-aminobutyric acid (GABAergic) neurons (145). Based on these studies, supplementation with melatonin has been considered as an effective method to alleviate sleep onset latency and to improve sleep quality in children (146) and adults (147, 148). Furthermore, the increase of melatonin secretion during sleep improves the sleep quality by enhancing the amplitude of circadian oscillations through melatonin receptors MT1 and MT2 (149).

Dysregulation of melatonin results in cognitive impairment by synaptic dysfunction. Recently, two studies have suggested that impairments of the sleep/wake cycle owing to sleep disturbances increase T2DM risk (128, 150). A genome-wide study revealed the relationship between single nucleotide polymorphisms in the *MTNR1B* gene (encoding MT2) and T2DM (151). In addition, decreased serum melatonin levels have been found in both diabetes mouse models and diabetes patients with hyperinsulinemia (152). Oral administration of melatonin alleviated hyperglycemia, hyperinsulinemia, and hyperlipidemia in T2DM rats (153, 154). Melatonin suppressed the levels of cytosolic cyclic adenosine monophosphate and/or cytosolic guanosine monophosphate and regulated insulin secretion via these receptors (155). Moreover, a study demonstrated that sleep disturbance in AD is related to the physiological changes in melatonin function (156). Furthermore, given that melatonin administration could attenuate the rate of AD progression, inhibit the accumulation of Aβ (157, 158), decrease neuronal cell death (159, 160), and reduce insulin resistance (152), the decrease in melatonin levels due to sleep disturbance might be associated with the impairment of the glymphatic system in diabetes-induced dementia. Diabetes triggers memory dysfunction in rats, which can be alleviated by melatonin treatment (161). Considering previous evidence, we suggest that melatonin administration should be considered as an approach to reduce neuropathology in diabetes-induced dementia.

A recent study demonstrated that impaired sleep duration was recorded in hyperglycemia patients (162). Epidemiological studies also showed that short duration and poor quality of sleep increase the risk of diabetes in adults (163, 164). Consequently, sleep impairment is strongly related to diabetes pathologies such as hyperglycemia (165).

The study reported that the lateral decubitus body position during sleep leads to an enhanced influx of a fluorescent CSF tracer into the cerebrum with a reduction of interstitial solute retention and an increase of clearance efficiency (43). Smooth glymphatic flow during sleep contributes to the improvement of paracrine signaling, whereas the decline in glymphatic flow suppresses the perivascular lipid transport, the astrocytic Ca^2+^ signaling within the cortex (30), and the opening of NMDA receptors (166). Moreover, a recent study suggested that AQP4-mediated glymphatic pathway improvement could be used as a therapeutic treatment for AD patients (167). The *AQP4* gene could regulate the progression of cognitive dysfunction in AD, and this was related to poor sleep and Aβ burden (168). The genetic variation in *AQP4* was also identified to be a factor correlating sleep and Aβ accumulation in the brain (169).

Based on the previous evidence stated, the sleep-induced metabolite clearance through the glymphatic system has a critical role in neuropathological features, including excessive accumulation of Aβ in brains with diabetes-induced dementia. Thus, we highlight that the improvement of glymphatic system function by the regulation of sleep may be a promising and effective strategy to reduce the neuropathological symptoms observed in diabetes-induced dementia.

## Conclusions

**Figure 1:**
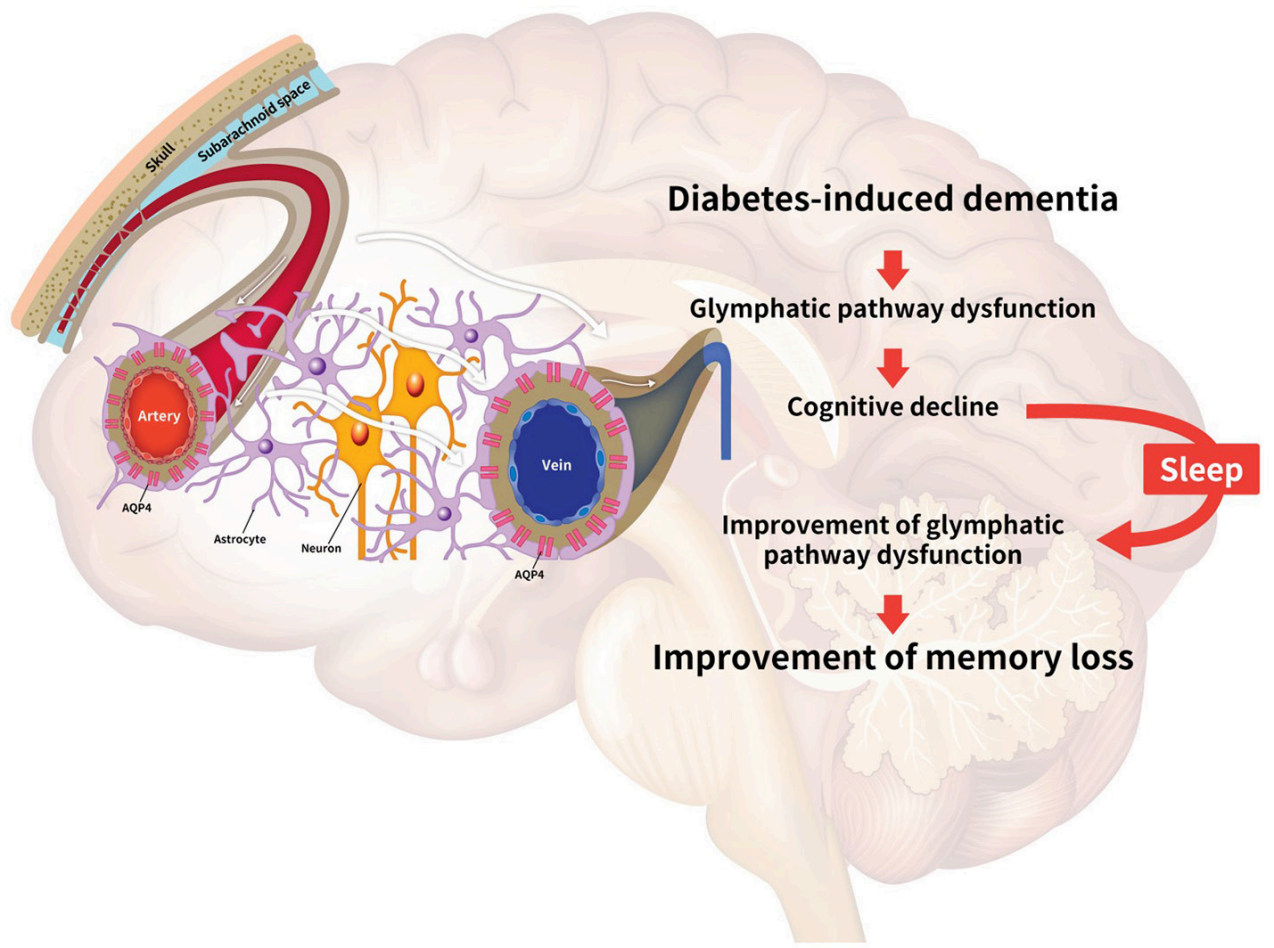
Schematic image illustrating the relationship between the glymphatic system and sleep. Diabetes conditions trigger the impairment of the glymphatic pathway. The decrease of glymphatic activity results in reduced efficiency for clearing toxic peptides, dysregulation of glucose metabolism, insulin resistance, and dysregulation of apolipoprotein E (APOE) circulation in the brain, leading to cognitive decline. Sleep improves the clearance of toxic amyloid-beta (Aβ), the secretion of norepinephrine, and the circulation of APOE and melatonin in the brain. Collectively, sleep is associated with the alleviation of the impaired glymphatic function, leading to the enhancement of memory in diabetes-induced dementia. The white arrows indicate the glymphatic flow.

In diabetes-induced dementia, the glymphatic system dysfunction characterized by the failure of interstitial solute clearance leads to extracellular solute accumulation and cognitive decline. Even though there is no experimental approach providing a direct relationship between sleep and the glymphatic system, many studies have implicated their relationship. Thus, we highlight the necessity for further exploration of the improvement of glymphatic system through sleep modulation toward attenuation of the neuropathology in diabetes-induced dementia. Here, we have reviewed the dysregulation of the glymphatic pathway in diabetes-induced dementia, the effects of sleep on glymphatic system function, including the improvement of toxic peptide clearance, the enhancement of melatonin secretion, the regulation of APOE expression, the improvement of synaptic plasticity, the regulation of norepinephrine levels, and the alleviation of insulin resistance (11, 120, 128, 130, 170, 171) (Figure 1).

Hence, we suggest that the improvement of glymphatic function by sleep regulation may be a novel target for attenuating neuropathological symptoms such as memory loss in diabetes-induced dementia, through the enhancement of the circulation of melatonin, APOE and norepinephrine, and reduction of Aβ aggregation in the brain.

## Author contributions

Y-KK and JS wrote the manuscript. JS and KN prepared the figure.

### Conflict of interest statement

The authors declare that the research was conducted in the absence of any commercial or financial relationships that could be construed as a potential conflict of interest.

## References

[1] Mittal K Katare DP. Shared links between type 2 diabetes mellitus and Alzheimer's disease: a review. Diabetes Metab Syndr. (2016) 10(2 Suppl. 1):S144–9. 10.1016/j.dsx.2016.01.021 26907971

[2] Gudala K Bansal D Schifano F Bhansali A. Diabetes mellitus and risk of dementia: a meta-analysis of prospective observational studies. J Diabetes Investig. (2013) 4:640–50. 10.1111/jdi.12087 24843720 PMC4020261

[3] Haroon NN Austin PC Shah BR Wu J Gill SS Booth GL. Risk of dementia in seniors with newly diagnosed diabetes: a population-based study. Diabetes Care (2015) 38:1868–75. 10.2337/dc15-0491 26216873

[4] Shaik MA Chan QL Xu J Xu X Hui RJ Chong SS. Risk factors of cognitive impairment and brief cognitive tests to predict cognitive performance determined by a formal neuropsychological evaluation of primary health care patients. J Am Med Dir Assoc. (2016) 17:343–7. 10.1016/j.jamda.2015.12.007 26785695

[5] McCrimmon RJ Ryan CM Frier BM. Diabetes and cognitive dysfunction. Lancet (2012) 379:2291–9. 10.1016/S0140-6736(12)60360-2 22683129

[6] Vanhanen M Koivisto K Kuusisto J Mykkanen L Helkala EL Hanninen T. Cognitive function in an elderly population with persistent impaired glucose tolerance. Diabetes Care (1998) 21:398–402. 10.2337/diacare.21.3.398 9540022

[7] Tarasoff-Conway JM Carare RO Osorio RS Glodzik L Butler T Fieremans E. Clearance systems in the brain-implications for Alzheimer disease. Nat Rev Neurol. (2015) 11:457–70. 10.1038/nrneurol.2015.119 26195256 PMC4694579

[8] Jessen NA Munk AS Lundgaard I Nedergaard M. The Glymphatic system: a beginner's guide. Neurochem Res. (2015) 40:2583–99. 10.1007/s11064-015-1581-6 25947369 PMC4636982

[9] Peng W Achariyar TM Li B Liao Y Mestre H Hitomi E. Suppression of glymphatic fluid transport in a mouse model of Alzheimer's disease. Neurobiol Dis. (2016) 93:215–25. 10.1016/j.nbd.2016.05.015 27234656 PMC4980916

[10] Moheet A Mangia S Seaquist ER. Impact of diabetes on cognitive function and brain structure. Ann N Y Acad Sci. (2015) 1353:60–71. 10.1111/nyas.12807 26132277 PMC4837888

[11] Yang G Lai CS Cichon J Ma L Li W Gan WB. Sleep promotes branch-specific formation of dendritic spines after learning. Science (2014) 344:1173–8. 10.1126/science.1249098 24904169 PMC4447313

[12] Baglietto-Vargas D Shi J Yaeger DM Ager R LaFerla FM. Diabetes and Alzheimer's disease crosstalk. Neurosci Biobehav Rev. (2016) 64:272–87. 10.1016/j.neubiorev.2016.03.005 26969101

[13] Kondo H Shirotake S Okabe T Makino S Nishimoto K Oyama M. Clinical impact of consolidative and salvage radiotherapy for lymph node metastasis in upper urinary tract urothelial carcinoma. Case Rep Urol. (2018) 2018:1471839. 10.1155/2018/1471839 29850366 PMC5937622

[14] Wardlaw JM Smith C Dichgans M. Mechanisms of sporadic cerebral small vessel disease: insights from neuroimaging. Lancet Neurol. (2013) 12:483–97. 10.1016/S1474-4422(13)70060-7 23602162 PMC3836247

[15] Xie L Kang H Xu Q Chen MJ Liao Y Thiyagarajan M. Sleep drives metabolite clearance from the adult brain. Science (2013) 342:373–7. 10.1126/science.1241224 24136970 PMC3880190

[16] Jackson ML Gunzelmann G Whitney P Hinson JM Belenky G Rabat A. Deconstructing and reconstructing cognitive performance in sleep deprivation. Sleep Med Rev. (2013) 17:215–25. 10.1016/j.smrv.2012.06.007 22884948 PMC3498579

[17] Iliff JJ Nedergaard M. Is there a cerebral lymphatic system? Stroke (2013) 44(6 Suppl. 1):S93–95. 10.1161/STROKEAHA.112.678698 23709744 PMC3699410

[18] Iliff JJ Wang M Liao Y Plogg BA Peng W Gundersen GA. A paravascular pathway facilitates CSF flow through the brain parenchyma and the clearance of interstitial solutes, including amyloid beta. Sci Transl Med. (2012) 4:147ra111. 10.1126/scitranslmed.3003748 22896675 PMC3551275

[19] Matsumae M Atsumi H Hirayama A Hayashi N Takizawa K Sano F. A new look at cerebrospinal fluid motion. No Shinkei Geka (2016) 44:909–24. 10.11477/mf.1436203402. 27832614

[20] Oreskovic D Klarica M. The formation of cerebrospinal fluid: nearly a hundred years of interpretations and misinterpretations. Brain Res Rev. (2010) 64:241–62. 10.1016/j.brainresrev.2010.04.006 20435061

[21] Brinker T Stopa E Morrison J Klinge P. A new look at cerebrospinal fluid circulation. Fluids Barriers CNS (2014) 11:10. 10.1186/2045-8118-11-10 24817998 PMC4016637

[22] Sykova E Nicholson C. Diffusion in brain extracellular space. Physiol Rev. (2008) 88:1277–340. 10.1152/physrev.00027.2007 18923183 PMC2785730

[23] Louveau A Smirnov I Keyes TJ Eccles JD Rouhani SJ Peske JD. Structural and functional features of central nervous system lymphatic vessels. Nature (2015) 523:337–41. 10.1038/nature14432 26030524 PMC4506234

[24] Abbott NJ. Evidence for bulk flow of brain interstitial fluid: significance for physiology and pathology. Neurochem Int. (2004) 45:545–52. 10.1016/j.neuint.2003.11.006 15186921

[25] Smith AJ Jin BJ Verkman AS. Muddying the water in brain edema? Trends Neurosci. (2015) 38:331–2. 10.1016/j.tins.2015.04.006 25980601 PMC4739497

[26] Jin BJ Smith AJ Verkman AS Spatial model of convective solute transport in brain extracellular space does not support a “glymphatic” mechanism. J Gen Physiol. (2016) 148:489–501. 10.1085/jgp.201611684 27836940 PMC5129742

[27] Iliff JJ Goldman SA Nedergaard M. Implications of the discovery of brain lymphatic pathways. Lancet Neurol. (2015) 14:977–9. 10.1016/S1474-4422(15)00221-5 26376966 PMC4655610

[28] Pien GW Pack AI Jackson N Maislin G Macones GA Schwab RJ. Risk factors for sleep-disordered breathing in pregnancy. Thorax (2014) 69:371–7. 10.1136/thoraxjnl-2012-202718 24262432 PMC6994201

[29] Iliff JJ Wang M Zeppenfeld DM Venkataraman A Plog BA Liao Y. Cerebral arterial pulsation drives paravascular CSF-interstitial fluid exchange in the murine brain. J Neurosci. (2013b) 33:18190–9. 10.1523/JNEUROSCI.1592-13.2013 24227727 PMC3866416

[30] Rangroo Thrane V Thrane AS Plog BA Thiyagarajan M Iliff JJ Deane R Paravascular microcirculation facilitates rapid lipid transport and astrocyte signaling in the brain. Sci Rep. (2013) 3:2582 10.1038/srep02582 24002448 PMC3761080

[31] Plog BA Dashnaw ML Hitomi E Peng W Liao Y Lou N. Biomarkers of traumatic injury are transported from brain to blood via the glymphatic system. J Neurosci. (2015) 35:518–26. 10.1523/JNEUROSCI.3742-14.2015 25589747 PMC4293408

[32] Eide PK Ringstad G. MRI with intrathecal MRI gadolinium contrast medium administration: a possible method to assess glymphatic function in human brain. Acta Radiol Open (2015) 4:2058460115609635. 10.1177/2058460115609635 26634147 PMC4652208

[33] Taoka T Masutani Y Kawai H Nakane T Matsuoka K Yasuno F. Evaluation of glymphatic system activity with the diffusion MR technique: diffusion tensor image analysis along the perivascular space (DTI-ALPS) in Alzheimer's disease cases. Jpn J Radiol. (2017) 35:172–8. 10.1007/s11604-017-0617-z 28197821

[34] Ma Q Ineichen BV Detmar M Proulx ST. Outflow of cerebrospinal fluid is predominantly through lymphatic vessels and is reduced in aged mice. Nat Commun. (2017) 8:1434. 10.1038/s41467-017-01484-6 29127332 PMC5681558

[35] Nedergaard M. Neuroscience. Garbage truck of the brain Science. Science (2013) 340:1529–30. 10.1126/science.1240514 23812703 PMC3749839

[36] Achariyar TM Li B Peng W Verghese PB Shi Y McConnell E. Glymphatic distribution of CSF-derived apoE into brain is isoform specific and suppressed during sleep deprivation. Mol Neurodegener. (2016) 11:74. 10.1186/s13024-016-0138-8 27931262 PMC5146863

[37] Hawkins RA Peterson DR Vina JR. The complementary membranes forming the blood-brain barrier. IUBMB Life (2002) 54:101–7. 10.1080/15216540214541 12489636

[38] Popescu BO Toescu EC Popescu LM Bajenaru O Muresanu DF Schultzberg M. Blood-brain barrier alterations in ageing and dementia. J Neurol Sci. (2009) 283:99–106. 10.1016/j.jns.2009.02.321 19264328

[39] Zhang CE Wong SM van de Haar HJ Staals J Jansen JF Jeukens CR. Blood-brain barrier leakage is more widespread in patients with cerebral small vessel disease. Neurology (2017) 88:426–32. 10.1212/WNL.0000000000003556 28031395

[40] Berridge CW Waterhouse BD. The locus coeruleus-noradrenergic system: modulation of behavioral state and state-dependent cognitive processes. Brain Res Brain Res Rev. (2003) 42:33–84. 10.1016/S0165-0173(03)00143-7 12668290

[41] Gannon M Che P Chen Y Jiao K Roberson ED Wang Q. Noradrenergic dysfunction in Alzheimer's disease. Front Neurosci. (2015) 9:220. 10.3389/fnins.2015.00220 26136654 PMC4469831

[42] Sironi L Guerrini U Tremoli E Miller I Gelosa P Lascialfari A. Analysis of pathological events at the onset of brain damage in stroke-prone rats: a proteomics and magnetic resonance imaging approach. J Neurosci Res. (2004) 78:115–22. 10.1002/jnr.20219 15372505

[43] Lee H Xie L Yu M Kang H Feng T Deane R. The effect of body posture on brain glymphatic transport. J Neurosci. (2015) 35:11034–44. 10.1523/JNEUROSCI.1625-15.2015 26245965 PMC4524974

[44] Grandner MA Patel NP Jean-Louis G Jackson N Gehrman PR Perlis ML. Sleep-related behaviors and beliefs associated with race/ethnicity in women. J Natl Med Assoc. (2013) 105:4–15. 10.1016/S0027-9684(15)30080-8 23862291 PMC3759527

[45] Iliff JJ Lee H Yu M Feng T Logan J Nedergaard M. Brain-wide pathway for waste clearance captured by contrast-enhanced MRI. J Clin Invest. (2013) 123:1299–309. 10.1172/JCI67677 23434588 PMC3582150

[46] Jack CRJr Knopman DS Jagust WJ Shaw LM Aisen PS Weiner MW. Hypothetical model of dynamic biomarkers of the Alzheimer's pathological cascade. Lancet Neurol. (2010) 9:119–28. 10.1016/S1474-4422(09)70299-6 20083042 PMC2819840

[47] Parashar A Mehta V Malairaman U. Type 2 diabetes mellitus is associated with social recognition memory deficit and altered dopaminergic neurotransmission in the amygdala. Ann Neurosci. (2018) 24:212–20. 10.1159/000479637 29849445 PMC5969354

[48] Al Khatib HK Harding SV Darzi J Pot GK The effects of partial sleep deprivation on energy balance: a systematic review and meta-analysis. Eur J Clin Nutr. (2017) 71:614–24. 10.1038/ejcn.2016.201 27804960

[49] Verheggen ICM Van Boxtel MPJ Verhey FRJ Jansen JFA Backes WH. Interaction between blood-brain barrier and glymphatic system in solute clearance. Neurosci Biobehav Rev. (2018) 90:26–33. 10.1016/j.neubiorev.2018.03.028 29608988

[50] Grandner MA Jackson N Gooneratne NS Patel NP. The development of a questionnaire to assess sleep-related practices, beliefs, and attitudes. Behav Sleep Med. (2014) 12:123–42. 10.1080/15402002.2013.764530 23514261 PMC3795978

[51] Selkoe DJ Hardy J. The amyloid hypothesis of Alzheimer's disease at 25 years. EMBO Mol Med. (2016) 8:595–608. 10.15252/emmm.201606210 27025652 PMC4888851

[52] Burgmans S van de Haar HJ Verhey FR Backes WH. Amyloid-beta interacts with blood-brain barrier function in dementia: a systematic review. J Alzheimers Dis. (2013) 35:859–73. 10.3233/JAD-122155 23542866

[53] Zuroff L Daley D Black KL Koronyo-Hamaoui M. Clearance of cerebral Abeta in Alzheimer's disease: reassessing the role of microglia and monocytes. Cell Mol Life Sci. (2017) 74:2167–201. 10.1007/s00018-017-2463-7 28197669 PMC5425508

[54] Kress BT Iliff JJ Xia M Wang M Wei HS Zeppenfeld D. Impairment of paravascular clearance pathways in the aging brain. Ann Neurol. (2014) 76:845–61. 10.1002/ana.24271 25204284 PMC4245362

[55] Simon MJ Iliff JJ. Regulation of cerebrospinal fluid (CSF) flow in neurodegenerative, neurovascular and neuroinflammatory disease. Biochim Biophys Acta (2016) 1862:442–51. 10.1016/j.bbadis.2015.10.014 26499397 PMC4755861

[56] Morris AW Carare RO Schreiber S Hawkes CA. The cerebrovascular basement membrane: role in the clearance of beta-amyloid and cerebral amyloid angiopathy. Front Aging Neurosci. (2014) 6:251. 10.3389/fnagi.2014.00251 25285078 PMC4168721

[57] van de Haar HJ Burgmans S Jansen JF van Osch MJ van Buchem MA Muller M Blood-brain barrier leakage in patients with early Alzheimer disease. Radiology (2016) 281:527–35. 10.1148/radiol.2016152244 27243267

[58] Wostyn P Killer HE De Deyn PP. Glymphatic stasis at the site of the lamina cribrosa as a potential mechanism underlying open-angle glaucoma. Clin Exp Ophthalmol. (2017) 45:539–47. 10.1111/ceo.12915 28129671

[59] Jiang Q Zhang L Ding G Davoodi-Bojd E Li Q Li L. Impairment of the glymphatic system after diabetes. J Cereb Blood Flow Metab. (2017) 37:1326–37. 10.1177/0271678X16654702 27306755 PMC5453454

[60] Venkat P Chopp M Zacharek A Cui C Zhang L Li Q. White matter damage and glymphatic dysfunction in a model of vascular dementia in rats with no prior vascular pathologies. Neurobiol Aging (2017) 50:96–106. 10.1016/j.neurobiolaging.2016.11.002 27940353 PMC5209254

[61] Lundgaard I Lu ML Yang E Peng W Mestre H Hitomi E. Glymphatic clearance controls state-dependent changes in brain lactate concentration. J Cereb Blood Flow Metab. (2017) 37:2112–24. 10.1177/0271678X16661202 27481936 PMC5464705

[62] Howard ME Jackson ML Berlowitz D O'Donoghue F Swann P Westlake J. Specific sleepiness symptoms are indicators of performance impairment during sleep deprivation. Accid Anal Prev. (2014) 62:1–8. 10.1016/j.aap.2013.09.003 24125802

[63] Zeppenfeld DM Simon M Haswell JD D'Abreo D Murchison C Quinn JF. Association of perivascular localization of aquaporin-4 with cognition and alzheimer disease in aging brains. JAMA Neurol. (2017) 74:91–9. 10.1001/jamaneurol.2016.4370 27893874

[64] Moftakhar P Lynch MD Pomakian JL Vinters HV Aquaporin expression in the brains of patients with or without cerebral amyloid angiopathy. J Neuropathol Exp Neurol. (2010) 69:1201–9. 10.1097/NEN.0b013e3181fd252c 21107133 PMC3155418

[65] Yang W Wu Q Yuan C Gao J Xiao M Gu M. Aquaporin-4 mediates astrocyte response to beta-amyloid. Mol Cell Neurosci. (2012) 49:406–14. 10.1016/j.mcn.2012.02.002 22365952

[66] Xu Z Xiao N Chen Y Huang H Marshall C Gao J. Deletion of aquaporin-4 in APP/PS1 mice exacerbates brain Abeta accumulation and memory deficits. Mol Neurodegener. (2015) 10:58. 10.1186/s13024-015-0056-1 26526066 PMC4631089

[67] Xiao M Hu G. Involvement of aquaporin 4 in astrocyte function and neuropsychiatric disorders. CNS Neurosci Ther. (2014) 20:385–90. 10.1111/cns.12267 24712483 PMC6493026

[68] Sorani MD Zador Z Hurowitz E Yan D Giacomini KM Manley GT. Novel variants in human Aquaporin-4 reduce cellular water permeability. Hum Mol Genet. (2008) 17:2379–89. 10.1093/hmg/ddn138 18511455 PMC2733814

[69] Skucas VA Mathews IB Yang J Cheng Q Treister A Duffy AM. Impairment of select forms of spatial memory and neurotrophin-dependent synaptic plasticity by deletion of glial aquaporin-4. J Neurosci. (2011) 31:6392–7. 10.1523/JNEUROSCI.6249-10.2011 21525279 PMC3107562

[70] Fan Y Liu M Wu X Wang F Ding J Chen J. Aquaporin-4 promotes memory consolidation in Morris water maze. Brain Struct Funct. (2013) 218:39–50. 10.1007/s00429-011-0373-2 22193336

[71] Zhang J Li Y Chen ZG Dang H Ding JH Fan Y. Glia protein aquaporin-4 regulates aversive motivation of spatial memory in Morris water maze. CNS Neurosci Ther. (2013) 19:937–44. 10.1111/cns.12191 24165567 PMC6493407

[72] Swanson LW Hartman BK. The central adrenergic system. An immunofluorescence study of the location of cell bodies and their efferent connections in the rat utilizing dopamine-beta-hydroxylase as a marker. J Comp Neurol. (1975) 163:467–505. 10.1002/cne.901630406 1100685

[73] Harik SI. Blood–brain barrier sodium/potassium pump: modulation by central noradrenergic innervation. Proc Natl Acad Sci USA. (1986) 83:4067–70. 10.1073/pnas.83.11.4067 3012548 PMC323667

[74] Chamberlain SR Robbins TW. Noradrenergic modulation of cognition: therapeutic implications. J Psychopharmacol. (2013) 27:694–718. 10.1177/0269881113480988 23518815

[75] Elrod R Peskind ER DiGiacomo L Brodkin KI Veith RC Raskind MA. Effects of Alzheimer's disease severity on cerebrospinal fluid norepinephrine concentration. Am J Psychiatry (1997) 154:25–30. 10.1176/ajp.154.1.25 8988954

[76] Zarow C Lyness SA Mortimer JA Chui HC. Neuronal loss is greater in the locus coeruleus than nucleus basalis and substantia nigra in Alzheimer and Parkinson diseases. Arch Neurol. (2003) 60:337–41. 10.1001/archneur.60.3.337 12633144

[77] Chen Y Peng Y Che P Gannon M Liu Y Li L. alpha(2A) adrenergic receptor promotes amyloidogenesis through disrupting APP-SorLA interaction. Proc Natl Acad Sci USA. (2014) 111:17296–301. 10.1073/pnas.1409513111 25404298 PMC4260556

[78] Wang D Fu Q Zhou Y Xu B Shi Q Igwe B. beta2 adrenergic receptor, protein kinase A (PKA) and c-Jun N-terminal kinase (JNK) signaling pathways mediate tau pathology in Alzheimer disease models. J Biol Chem. (2013) 288:10298–307. 10.1074/jbc.M112.415141 23430246 PMC3624413

[79] Schmechel DE Saunders AM Strittmatter WJ Crain BJ Hulette CM Joo SH. Increased amyloid beta-peptide deposition in cerebral cortex as a consequence of apolipoprotein E genotype in late-onset Alzheimer disease. Proc Natl Acad Sci USA. (1993) 90:9649–53. 10.1073/pnas.90.20.9649 8415756 PMC47627

[80] Dallongeville J Lussier-Cacan S Davignon J. Modulation of plasma triglyceride levels by apoE phenotype: a meta-analysis. J Lipid Res. (1992) 33:447–54. 1388198

[81] Liu CC Liu CC Kanekiyo T Xu H Bu G. Apolipoprotein E and Alzheimer disease: risk, mechanisms and therapy. Nat Rev Neurol. (2013) 9:106–18. 10.1038/nrneurol.2012.263 23296339 PMC3726719

[82] Iliff JJ Chen MJ Plog BA Zeppenfeld DM Soltero M Yang L. Impairment of glymphatic pathway function promotes tau pathology after traumatic brain injury. J Neurosci. (2014) 34:16180–93. 10.1523/JNEUROSCI.3020-14.2014 25471560 PMC4252540

[83] Mahley RW Rall SCJr. Apolipoprotein E: far more than a lipid transport protein. Annu Rev Genomics Hum Genet. (2000) 1:507–37. 10.1146/annurev.genom.1.1.507 11701639

[84] Huang Y Weisgraber KH Mucke L Mahley RW. Apolipoprotein E: diversity of cellular origins, structural and biophysical properties, and effects in Alzheimer's disease. J Mol Neurosci. (2004) 23:189–204. 10.1385/JMN:23:3:189 15181247

[85] Hatters DM Zhong N Rutenber E Weisgraber KH. Amino-terminal domain stability mediates apolipoprotein E aggregation into neurotoxic fibrils. J Mol Biol. (2006) 361:932–44. 10.1016/j.jmb.2006.06.080 16890957

[86] Verghese PB Castellano JM Holtzman DM. Apolipoprotein E in Alzheimer's disease and other neurological disorders. Lancet Neurol. (2011) 10:241–52. 10.1016/S1474-4422(10)70325-2 21349439 PMC3132088

[87] Giorda CB Avogaro A Maggini M Lombardo F Mannucci E Turco S. Incidence and risk factors for stroke in type 2 diabetic patients: the DAI study. Stroke (2007) 38:1154–60. 10.1161/01.STR.0000260100.71665.2f 17332448

[88] Janghorbani M Hu FB Willett WC Li TY Manson JE Logroscino G. Prospective study of type 1 and type 2 diabetes and risk of stroke subtypes: the Nurses' Health Study. Diabetes Care (2007) 30:1730–5. 10.2337/dc06-2363 17389335

[89] Dallongeville J Selinger E Davignon J Lussier-Cacan S. Fish-oil supplementation reduces Ip(a) concentrations in type III dysbetalipoproteinemia. Clin Chem. (1992) 38(8 Pt 1), 1510–1. 1386561

[90] Corder EH Saunders AM Strittmatter WJ Schmechel DE Gaskell PC Small GW. Gene dose of apolipoprotein E type 4 allele and the risk of Alzheimer's disease in late onset families. Science (1993) 261:921–3. 10.1126/science.8346443 8346443

[91] Strittmatter WJ Saunders AM Schmechel D Pericak-Vance M Enghild J Salvesen GS. Apolipoprotein E: high-avidity binding to beta-amyloid and increased frequency of type 4 allele in late-onset familial Alzheimer disease. Proc Natl Acad Sci USA. (1993) 90:1977–81. 10.1073/pnas.90.5.1977 8446617 PMC46003

[92] Pintana H Apaijai N Kerdphoo S Pratchayasakul W Sripetchwandee J Suntornsaratoon P. Hyperglycemia induced the Alzheimer's proteins and promoted loss of synaptic proteins in advanced-age female Goto-Kakizaki (GK) rats. Neurosci Lett. (2017) 655:41–5. 10.1016/j.neulet.2017.06.041 28652187

[93] Giwa MO Williams J Elderfield K Jiwa NS Bridges LR Kalaria RN. Neuropathologic evidence of endothelial changes in cerebral small vessel disease. Neurology (2012) 78:167–74. 10.1212/WNL.0b013e3182407968 22170884

[94] Prasad S Sajja RK Naik P Cucullo L. Diabetes mellitus and blood-brain barrier dysfunction: an overview. J Pharmacovigil. (2014) 2:125. 10.4172/2329-6887.1000125 25632404 PMC4306190

[95] Ho L Qin W Pompl PN Xiang Z Wang J Zhao Z. Diet-induced insulin resistance promotes amyloidosis in a transgenic mouse model of Alzheimer's disease. FASEB J. (2004) 18:902–4. 10.1096/fj.03-0978fje 15033922

[96] Yang Y Wu Y Zhang S Song W. High glucose promotes Abeta production by inhibiting APP degradation. PLoS ONE (2013) 8:e69824. 10.1371/journal.pone.0069824 23894546 PMC3720941

[97] Mehla J Chauhan BC Chauhan NB. Experimental induction of type 2 diabetes in aging-accelerated mice triggered Alzheimer-like pathology and memory deficits. J Alzheimers Dis. (2014) 39:145–62. 10.3233/JAD-131238 24121970 PMC3941701

[98] Vandal M White PJ Tremblay C St-Amour I Chevrier G Emond V. Insulin reverses the high-fat diet-induced increase in brain Abeta and improves memory in an animal model of Alzheimer disease. Diabetes (2014) 63:4291–301. 10.2337/db14-0375 25008180

[99] Mayeda ER Whitmer RA Yaffe K. Diabetes and cognition. Clin Geriatr Med. (2015) 31:101–15, ix. 10.1016/j.cger.2014.08.021 25453304 PMC4370221

[100] Qiu C Sigurdsson S Zhang Q Jonsdottir MK Kjartansson O Eiriksdottir G. Diabetes, markers of brain pathology and cognitive function: the Age, gene/environment susceptibility-reykjavik study. Ann Neurol. (2014) 75:138–46. 10.1002/ana.24063 24243491 PMC4540233

[101] Luchsinger JA Gustafson DR. Adiposity, type 2 diabetes, and Alzheimer's disease. J Alzheimers Dis. (2009) 16:693–704. 10.3233/JAD-2009-1022 19387106 PMC2705908

[102] Doubal FN MacLullich AM Ferguson KJ Dennis MS Wardlaw JM. Enlarged perivascular spaces on MRI are a feature of cerebral small vessel disease. Stroke (2010) 41:450–4. 10.1161/STROKEAHA.109.564914 20056930

[103] Acharya NK Levin EC Clifford PM Han M Tourtellotte R Chamberlain D. Diabetes and hypercholesterolemia increase blood-brain barrier permeability and brain amyloid deposition: beneficial effects of the LpPLA2 inhibitor darapladib. J Alzheimers Dis. (2013) 35:179–98. 10.3233/JAD-122254 23388174

[104] Hawkins BT Davis TP. The blood-brain barrier/neurovascular unit in health and disease. Pharmacol Rev. (2005) 57:173–85. 10.1124/pr.57.2.4 15914466

[105] Zlokovic BV. The blood-brain barrier in health and chronic neurodegenerative disorders. Neuron (2008) 57:178–201. 10.1016/j.neuron.2008.01.003 18215617

[106] Giacco F Brownlee M. Oxidative stress and diabetic complications. Circ Res. (2010) 107:1058–70. 10.1161/CIRCRESAHA.110.223545 21030723 PMC2996922

[107] Huang J Agus DB Winfree CJ Kiss S Mack WJ McTaggart RA. Dehydroascorbic acid, a blood-brain barrier transportable form of vitamin C, mediates potent cerebroprotection in experimental stroke. Proc Natl Acad Sci USA. (2001) 98:11720–4. 10.1073/pnas.171325998 11573006 PMC58796

[108] Minamizono A Tomi M Hosoya K. Inhibition of dehydroascorbic acid transport across the rat blood-retinal and -brain barriers in experimental diabetes. Biol Pharm Bull. (2006) 29:2148–50. 10.1248/bpb.29.2148 17015969

[109] Prakash R Johnson M Fagan SC Ergul A. Cerebral neovascularization and remodeling patterns in two different models of type 2 diabetes. PLoS ONE (2013) 8:e56264. 10.1371/journal.pone.0056264 23441170 PMC3575336

[110] Russo VC Higgins S Werther GA Cameron FJ. Effects of fluctuating glucose levels on neuronal cells in vitro. Neurochem Res. (2012) 37:1768–82. 10.1007/s11064-012-0789-y 22565596

[111] Haorah J Ramirez SH Schall K Smith D Pandya R Persidsky Y. Oxidative stress activates protein tyrosine kinase and matrix metalloproteinases leading to blood-brain barrier dysfunction. J Neurochem. (2007) 101:566–76. 10.1111/j.1471-4159.2006.04393.x 17250680

[112] Brownlee M. Biochemistry and molecular cell biology of diabetic complications. Nature (2001) 414:813–20. 10.1038/414813a 11742414

[113] Cipolla MJ Huang Q Sweet JG. Inhibition of protein kinase Cbeta reverses increased blood-brain barrier permeability during hyperglycemic stroke and prevents edema formation in vivo. Stroke (2011) 42:3252–7. 10.1161/STROKEAHA.111.623991 21852606 PMC3202059

[114] Peila R Rodriguez BL Launer LJ Honolulu-Asia Aging S. Type 2 diabetes, APOE gene, and the risk for dementia and related pathologies: the Honolulu-Asia aging study. Diabetes (2002) 51:1256–62. 10.2337/diabetes.51.4.1256 11916953

[115] Sasaki N Fukatsu R Tsuzuki K Hayashi Y Yoshida T Fujii N. Advanced glycation end products in Alzheimer's disease and other neurodegenerative diseases. Am J Pathol. (1998) 153:1149–55. 10.1016/S0002-9440(10)65659-3 9777946 PMC1853056

[116] Cirelli C Gutierrez CM Tononi G. Extensive and divergent effects of sleep and wakefulness on brain gene expression. Neuron (2004) 41:35–43. 10.1016/S0896-6273(03)00814-6 14715133

[117] Aton SJ Seibt J Dumoulin M Jha SK Steinmetz N Coleman T. Mechanisms of sleep-dependent consolidation of cortical plasticity. Neuron (2009) 61:454–66. 10.1016/j.neuron.2009.01.007 19217381 PMC2665998

[118] Tononi G Cirelli C. Sleep and the price of plasticity: from synaptic and cellular homeostasis to memory consolidation and integration. Neuron (2014) 81:12–34. 10.1016/j.neuron.2013.12.025 24411729 PMC3921176

[119] Lucke-Wold BP Smith KE Nguyen L Turner RC Logsdon AF Jackson GJ. Sleep disruption and the sequelae associated with traumatic brain injury. Neurosci Biobehav Rev. (2015) 55:68–77. 10.1016/j.neubiorev.2015.04.010 25956251 PMC4721255

[120] Bird SM Sohrabi HR Sutton TA Weinborn M Rainey-Smith SR Brown B. Cerebral amyloid-beta accumulation and deposition following traumatic brain injury–a narrative review and meta-analysis of animal studies. Neurosci Biobehav Rev. (2016) 64:215–28. 10.1016/j.neubiorev.2016.01.004 26899257

[121] Binder S Baier PC Molle M Inostroza M Born J Marshall L. Sleep enhances memory consolidation in the hippocampus-dependent object-place recognition task in rats. Neurobiol Learn Mem. (2012) 97:213–9. 10.1016/j.nlm.2011.12.004 22207008

[122] Acosta-Pena E Camacho-Abrego I Melgarejo-Gutierrez M Flores G Drucker-Colin R Garcia-Garcia F. Sleep deprivation induces differential morphological changes in the hippocampus and prefrontal cortex in young and old rats. Synapse (2015) 69:15–25. 10.1002/syn.21779 25179486

[123] Kyrtsos CR Baras JS. Modeling the role of the glymphatic pathway and cerebral blood vessel properties in alzheimer's disease pathogenesis. PLoS ONE (2015) 10:e0139574. 10.1371/journal.pone.0139574 26448331 PMC4598011

[124] Branger P Arenaza-Urquijo EM Tomadesso C Mezenge F Andre C de Flores R. Relationships between sleep quality and brain volume, metabolism, and amyloid deposition in late adulthood. Neurobiol Aging (2016) 41:107–14. 10.1016/j.neurobiolaging.2016.02.009 27103523

[125] Brown BM Rainey-Smith SR Villemagne VL Weinborn M Bucks RS Sohrabi HR. The relationship between sleep quality and brain amyloid burden. Sleep (2016) 39:1063–8. 10.5665/sleep.5756 27091528 PMC4835305

[126] Vanderburg CR Davis DA Diamond RE Kao PF Delalle I. Capzb2 protein expression in the brains of patients diagnosed with alzheimer's disease and huntington's disease. Transl Neurosci. (2010) 1:55–8. 10.2478/v10134-010-0008-9 29662700 PMC5899514

[127] Cedernaes J Lampola L Axelsson EK Liethof L Hassanzadeh S Yeganeh A A single night of partial sleep loss impairs fasting insulin sensitivity but does not affect cephalic phase insulin release in young men. J Sleep Res. (2016) 25:5–10. 10.1111/jsr.12340 26361380

[128] Qian J Scheer F. Circadian system and glucose metabolism: implications for physiology and disease. Trends Endocrinol Metab. (2016) 27:282–93. 10.1016/j.tem.2016.03.005 27079518 PMC4842150

[129] Nedeltcheva AV Scheer FA. Metabolic effects of sleep disruption, links to obesity and diabetes. Curr Opin Endocrinol Diabetes Obes. (2014) 21:293–8. 10.1097/MED.0000000000000082 24937041 PMC4370346

[130] Hipolide DC Moreira KM Barlow KB Wilson AA Nobrega JN Tufik S. Distinct effects of sleep deprivation on binding to norepinephrine and serotonin transporters in rat brain. Prog Neuropsychopharmacol Biol Psychiatry (2005) 29:297–303. 10.1016/j.pnpbp.2004.11.015 15694238

[131] Longordo F Kopp C Luthi A. Consequences of sleep deprivation on neurotransmitter receptor expression and function. Eur J Neurosci. (2009) 29:1810–9. 10.1111/j.1460-9568.2009.06719.x 19492440

[132] Figlewicz DP Brot MD McCall AL Szot P. Diabetes causes differential changes in CNS noradrenergic and dopaminergic neurons in the rat: a molecular study. Brain Res. (1996) 736:54–60. 10.1016/0006-8993(96)00727-5 8930308

[133] Umhau JC Petrulis SG Diaz R Rawlings R George DT. Blood glucose is correlated with cerebrospinal fluid neurotransmitter metabolites. Neuroendocrinology (2003) 78:339–43. 10.1159/000074887 14688447

[134] Straznicky NE Grima MT Sari CI Eikelis N Lambert EA Nestel PJ. Neuroadrenergic dysfunction along the diabetes continuum: a comparative study in obese metabolic syndrome subjects. Diabetes (2012) 61:2506–16. 10.2337/db12-0138 22664956 PMC3447913

[135] Cipolla MJ Li R Vitullo L. Perivascular innervation of penetrating brain parenchymal arterioles. J Cardiovasc Pharmacol. (2004) 44:1–8. 10.1097/00005344-200407000-00001 15175551

[136] Tan DX Manchester LC Fuentes-Broto L Paredes SD Reiter RJ. Significance and application of melatonin in the regulation of brown adipose tissue metabolism: relation to human obesity. Obes Rev. (2011) 12:167–88. 10.1111/j.1467-789X.2010.00756.x 20557470

[137] Kumar Jha P Challet E Kalsbeek A Circadian rhythms in glucose and lipid metabolism in nocturnal and diurnal mammals. Mol Cell Endocrinol. (2015) 418(Pt 1):74–88. 10.1016/j.mce.2015.01.024 25662277

[138] Cajochen C Kräuchi K Wirz-Justice A. Role of melatonin in the regulation of human circadian rhythms and sleep. J Neuroendocrinol. (2003) 15:432–7. 10.1046/j.1365-2826.2003.00989.x 12622846

[139] El-Sherif Y Tesoriero J Hogan MV Wieraszko A. Melatonin regulates neuronal plasticity in the hippocampus. J Neurosci Res. (2003) 72:454–60. 10.1002/jnr.10605 12704807

[140] Bob P Fedor-Freybergh P. Melatonin, consciousness, and traumatic stress. J Pineal Res. (2008) 44:341–7. 10.1111/j.1600-079X.2007.00540.x 18410583

[141] Wang LM Suthana NA Chaudhury D Weaver DR Colwell CS. Melatonin inhibits hippocampal long-term potentiation. Eur J Neurosci. (2005) 22:2231–7. 10.1111/j.1460-9568.2005.04408.x 16262661 PMC2581482

[142] Ozcan M Yilmaz B Carpenter DO. Effects of melatonin on synaptic transmission and long-term potentiation in two areas of mouse hippocampus. Brain Res. (2006) 1111:90–4. 10.1016/j.brainres.2006.06.117 16919244

[143] Vanecek J Klein DC Melatonin inhibition of GnRH-induced LH release from neonatal rat gonadotroph: involvement of Ca2+ not cAMP. Am J Physiol. (1995) 269(1 Pt 1):E85–90. 10.1152/ajpendo.1995.269.1.E85 7631782

[144] Escames G Macias M Leon J Garcia J Khaldy H Martin M. Calcium-dependent effects of melatonin inhibition of glutamatergic response in rat striatum. J Neuroendocrinol. (2001) 13:459–66. 10.1046/j.1365-2826.2001.00656.x 11328457

[145] Cheng XP Sun H Ye ZY Zhou JN. Melatonin modulates the GABAergic response in cultured rat hippocampal neurons. J Pharmacol Sci. (2012) 119:177–85. 10.1254/jphs.11183FP 22673185

[146] Chang YS Lin MH Lee JH Lee PL Dai YS Chu KH. Melatonin supplementation for children with atopic dermatitis and sleep disturbance: a randomized clinical trial. JAMA Pediatr. (2016) 170:35–42. 10.1001/jamapediatrics.2015.3092 26569624

[147] Lahteenmaki R Puustinen J Vahlberg T Lyles A Neuvonen PJ Partinen M. Melatonin for sedative withdrawal in older patients with primary insomnia: a randomized double-blind placebo-controlled trial. Br J Clin Pharmacol. (2014) 77:975–85. 10.1111/bcp.12294 24286360 PMC4093923

[148] Amstrup AK Sikjaer T Mosekilde L Rejnmark L. The effect of melatonin treatment on postural stability, muscle strength, and quality of life and sleep in postmenopausal women: a randomized controlled trial. Nutr J. (2015) 14:102. 10.1186/s12937-015-0093-1 26424587 PMC4590707

[149] Touitou Y Bogdan A. Promoting adjustment of the sleep-wake cycle by chronobiotics. Physiol Behav. (2007) 90:294–300. 10.1016/j.physbeh.2006.09.001 17056076

[150] Shan Z Ma H Xie M Yan P Guo Y Bao W. Sleep duration and risk of type 2 diabetes: a meta-analysis of prospective studies. Diabetes Care (2015) 38:529–37. 10.2337/dc14-2073 25715415

[151] Gaulton KJ Ferreira T Lee Y Raimondo A Magi R Reschen ME. Genetic fine mapping and genomic annotation defines causal mechanisms at type 2 diabetes susceptibility loci. Nat Genet. (2015) 47:1415–25. 10.1038/ng.3437 26551672 PMC4666734

[152] Peschke E Frese T Chankiewitz E Peschke D Preiss U Schneyer U. Diabetic Goto Kakizaki rats as well as type 2 diabetic patients show a decreased diurnal serum melatonin level and an increased pancreatic melatonin-receptor status. J Pineal Res. (2006) 40:135–43. 10.1111/j.1600-079X.2005.00287.x 16441550

[153] Rios-Lugo MJ Cano P Jimenez-Ortega V Fernandez-Mateos MP Scacchi PA Cardinali DP. Melatonin effect on plasma adiponectin, leptin, insulin, glucose, triglycerides and cholesterol in normal and high fat-fed rats. J Pineal Res. (2010) 49:342–8. 10.1111/j.1600-079X.2010.00798.x 20663045

[154] Agil A Rosado I Ruiz R Figueroa A Zen N Fernandez-Vazquez G. Melatonin improves glucose homeostasis in young Zucker diabetic fatty rats. J Pineal Res. (2012) 52:203–10. 10.1111/j.1600-079X.2011.00928.x 21883445

[155] Peschke E Bahr I Muhlbauer E. Melatonin and pancreatic islets: interrelationships between melatonin, insulin and glucagon. Int J Mol Sci. (2013) 14:6981–7015. 10.3390/ijms14046981 23535335 PMC3645673

[156] Wu YH Swaab DF. Disturbance and strategies for reactivation of the circadian rhythm system in aging and Alzheimer's disease. Sleep Med. (2007) 8:623–36. 10.1016/j.sleep.2006.11.010 17383938

[157] Poeggeler B Miravalle L Zagorski MG Wisniewski T Chyan YJ Zhang Y. Melatonin reverses the profibrillogenic activity of apolipoprotein E4 on the Alzheimer amyloid Abeta peptide. Biochemistry (2001) 40:14995–5001. 10.1021/bi0114269 11732920

[158] Francioso A Punzi P Boffi A Lori C Martire S Giordano C. beta-sheet interfering molecules acting against beta-amyloid aggregation and fibrillogenesis. Bioorg Med Chem. (2015) 23:1671–83. 10.1016/j.bmc.2015.02.041 25769517

[159] Blask DE Brainard GC Dauchy RT Hanifin JP Davidson LK Krause JA. Melatonin-depleted blood from premenopausal women exposed to light at night stimulates growth of human breast cancer xenografts in nude rats. Cancer Res. (2005) 65:11174–84. 10.1158/0008-5472.CAN-05-1945 16322268

[160] Stevens RG. Light-at-night, circadian disruption and breast cancer: assessment of existing evidence. Int J Epidemiol. (2009) 38:963–70. 10.1093/ije/dyp178 19380369 PMC2734067

[161] Babaei-Balderlou F Zare S. Melatonin improves spatial navigation memory in male diabetic rats. Vet Res Forum. (2012) 3:187–92. 25610567 PMC4299981

[162] DePietro RH Knutson KL Spampinato L Anderson SL Meltzer DO Van Cauter E. Association between inpatient sleep loss and hyperglycemia of hospitalization. Diabetes Care (2017) 40:188–93. 10.2337/dc16-1683 27903614 PMC5250691

[163] Knutson KL Ryden AM Mander BA Van Cauter E. Role of sleep duration and quality in the risk and severity of type 2 diabetes mellitus. Arch Intern Med. (2006) 166:1768–74. 10.1001/archinte.166.16.1768 16983057

[164] Anothaisintawee T Reutrakul S Van Cauter E Thakkinstian A. Sleep disturbances compared to traditional risk factors for diabetes development: systematic review and meta-analysis. Sleep Med Rev. (2016) 30:11–24. 10.1016/j.smrv.2015.10.002 26687279

[165] Vgontzas AN Liao D Pejovic S Calhoun S Karataraki M Bixler EO. Insomnia with objective short sleep duration is associated with type 2 diabetes: a population-based study. Diabetes Care (2009) 32:1980–5. 10.2337/dc09-0284 19641160 PMC2768214

[166] Maneshi MM Maki B Gnanasambandam R Belin S Popescu GK Sachs F. Mechanical stress activates NMDA receptors in the absence of agonists. Sci Rep. (2017) 7:39610. 10.1038/srep39610 28045032 PMC5206744

[167] Yin M Pu T Wang L Marshall C Wu T Xiao M. Astroglial water channel aquaporin 4-mediated glymphatic clearance function: a determined factor for time-sensitive treatment of aerobic exercise in patients with Alzheimer's disease. Med Hypotheses (2018) 119:18–21. 10.1016/j.mehy.2018.07.016 30122483

[168] Burfeind KG Murchison CF Westaway SK Simon MJ Erten-Lyons D Kaye JA. The effects of noncoding aquaporin-4 single-nucleotide polymorphisms on cognition and functional progression of Alzheimer's disease. Alzheimers Dement (N Y) (2017) 3:348–59. 10.1016/j.trci.2017.05.001 29067342 PMC5651426

[169] Rainey-Smith SR Mazzucchelli GN Villemagne VL Brown BM Porter T Weinborn M. Genetic variation in Aquaporin-4 moderates the relationship between sleep and brain Abeta-amyloid burden. Transl Psychiatry (2018) 8:47. 10.1038/s41398-018-0094-x 29479071 PMC5865132

[170] Kiviniemi V Wang X Korhonen V Keinanen T Tuovinen T Autio J. Ultra-fast magnetic resonance encephalography of physiological brain activity - glymphatic pulsation mechanisms? J Cereb Blood Flow Metab. (2016) 36:1033–45. 10.1177/0271678X15622047 26690495 PMC4908626

[171] van den Berg R Mook-Kanamori DO Donga E van Dijk M van Dijk JG Lammers GJ. A single night of sleep curtailment increases plasma acylcarnitines: Novel insights in the relationship between sleep and insulin resistance. Arch Biochem Biophys. (2016) 589:145–51. 10.1016/j.abb.2015.09.017 26393786

